# Probiotic consumption and hepatic steatosis: results from the NHANES 2011–2016 and Mendelian randomization study

**DOI:** 10.3389/fnut.2024.1334935

**Published:** 2024-04-08

**Authors:** Yancheng Song, Wencong Guo, Junke Wang, Shuguang Liu, Zhaopeng Li, Yu Li

**Affiliations:** ^1^Department of Gastrointestinal Surgery, The Affiliated Hospital of Qingdao University, Qingdao, China; ^2^Department of Colorectal Surgery, The Sixth Affiliated Hospital of Sun Yat-sen University, Guangzhou, China; ^3^Institute of Nephrology, Zhong Da Hospital, Southeast University School of Medicine, Nanjing, China; ^4^Laboratory of Nephrology & Department of Nephrology, The Affiliated Qingdao Municipal Hospital of Qingdao University, Qingdao, China; ^5^Department of Cardiology, Qingdao Hiser Hospital Affiliated to Qingdao University, Qingdao, China

**Keywords:** probiotic, yogurt, hepatic steatosis, MAFLD, population-based study, NHANES, Mendelian randomization study

## Abstract

**Background:**

Recent research showed that probiotics treatment may reduce insulin resistance, regulate lipid metabolism, raise liver enzyme levels, and ameliorate inflammation in individuals with metabolic associated fatty liver disease (MAFLD). However, the possible effects of probiotic use on the progression of hepatic steatosis (HS) have not been identified. The purpose of this study was to investigate this in a large population database.

**Methods:**

The cross-sectional research was conducted among adults with complete data on probiotic yogurt consumption and HS in the 2011–2016 National Health and Nutrition Examination Survey (NHANES). Probiotic yogurt consumption was assessed using a dietary supplement questionnaire, while HS was evaluated with HS index (HSI). To explore their relationship, weighted univariate regression analysis, subgroup analysis, and interaction analysis were conducted. To evaluate the causal association between yogurt consumption and NAFLD, mendelian randomization analysis (MR) were performed. A restricted cubic spline (RCS) was used to analyze the relationship curve between the leves of yogurt consumption and hepatic steatosis.

**Results:**

A total of 7,891 participants were included in the study represented 146.7 million non-institutionalized residents of the United States, of whom 4,322 (54.77%) were diagnosed with HS. Multivariable logistic regression showed probiotic yogurt consumption had significantly inverse relationship for HS (OR = 0.84, 95% CI: 0.72–0.97, *p* = 0.02) after adjusting for all covariates. Once more, the independent relationship between probiotic yogurt consumption and HS was verified by subgroup analysis and interaction analysis. The MR analysis results indicate that there is no causal relationship between yogurt consumption and NAFLD. The RCS model demonstrated a robust J-shaped link between yogurt consumption and HS, revealing a significant decrease in risk within the lower range of yogurt consumption, which attained the lowest risk close to 0.4 cup.

**Conclusion:**

According to the NHANES data, the consumption of probiotics and yogurt has a beneficial effect on HS, whereas the MR results indicated it was not related to NAFLD. The RCS analysis indicates a J-shaped relationship between yogurt consumption and HS, which may account for the inconsistency in the results. Based on these findings, we recommend that adults take half a cup of yogurt daily.

## 1 Introduction

Hepatic steatosis (HS), the build-up of fat in the liver that is frequently associated with obesity, can progress to fibrosis and cirrhosis of the liver (1). Given the increasing prevalence of obesity worldwide, the deleterious effects of HS are becoming a growing challenge for public health (2). Metabolic associated fatty liver disease (MAFLD) is the most common progression of HS, affecting 30–40% of male and 15–20% of female in the general population (3). It is recognized as a hepatic manifestation of metabolic syndrome and is linked to insulin resistance, atherosclerosis, obesity, dyslipidemia, and hypertension. Currently, MAFLD is recognized as a systemic metabolic disorder, reflecting its extensive impact beyond the liver, including effects on cardiovascular health, endocrine function, and metabolic regulation (4). Consequently, high clinical attention should be paid to HS. To reduce the increasing public burden of these disorders, it will be critical to develop and implement effective therapeutic approaches that improve HS.

Probiotics, prebiotics, and synbiotics are becoming more widely available as people’s living standards improve. Prebiotics are nondigestible substrates that can selectively increase the development of beneficial living microorganisms in the gastrointestinal (5). Probiotics are living microorganisms that, when taken in sufficient proportions, provide health advantages on the host. They are accessible as nutritional supplements and foodstuffs such as fermented dairy products and fermented vegetables (6). Synbiotics are a mixture of living organisms (e.g., probiotics) and substrates (e.g., prebiotics) that are selectively utilized by host microorganisms and confer a health benefit on the host (7).

Numerous animal studies have shown that probiotics and prebiotics have great potential for treating liver illnesses by influencing intestinal microbes. The time, composition, and dosage of probiotics in clinical therapy should be investigated further (8). Prebiotics are more stable and safer to employ in the treatment of individuals with chronic liver disease (9). However, there have not been many prebiotics explored or utilized (10, 11).

Metabolic disorders, caused by intestinal microbial dysregulation, also played an important role in the pathogenesis of MAFLD (12, 13). Although there have been systematic reviews on the use of probiotics and prebiotics in the treatment and prevention of MAFLD, the evidence currently available is insufficient to decisively establish the benefits of probiotics on HS (14, 15). These are due in part to the restrictions of small sample sizes, dosage and strain heterogeneity, and differences in intervention duration. Furthermore, few studies have investigated the connection between probiotic foods that people regularly intake (rather than as supplements) and HS (16). Mendelian randomization (MR) is a statistical method for making causal conclusions in epidemiology etiology. By using instrumental variables as genetic predictors, frequent confounders such as the environment, socioeconomic circumstances, and individual behaviors are not able to influence the correlation of genes with illnesses (17). To address some of the problems raised in previous investigations, we used a two-sample MR analysis to assess the casual relationship between probiotic yogurt consumption and HS. Therefore, we aimed to perform a well-controlled, large population-based study to obtain a better understanding of the regulatory effects of probiotics in HS. In the present study, we used a nationwide, large population-based database to test the hypothesis that probiotic yogurt consumption is inversely associated with the prevalence or severity of HS.

## 2 Subjects and methods

### 2.1 Study population and survey

All data for this study were collected from the 2011–2016 cross-sectional survey of US National Health and Nutrition Examination Survey (NHANES). The NHANES is a cross-sectional study conducted by the National Center for Health Statistics (NCHS) to collect information about the health, diet, and nutritional status of the non-institutionalized civilian population in the US. The survey was approved by the US Research Ethics Review Board of the National Center for Health Statistics, ensuring informed consent from all participants. Thus, approval from other ethics committees was not necessary in this study. All data are freely available in NHANES.^1^

**Figure 1:**
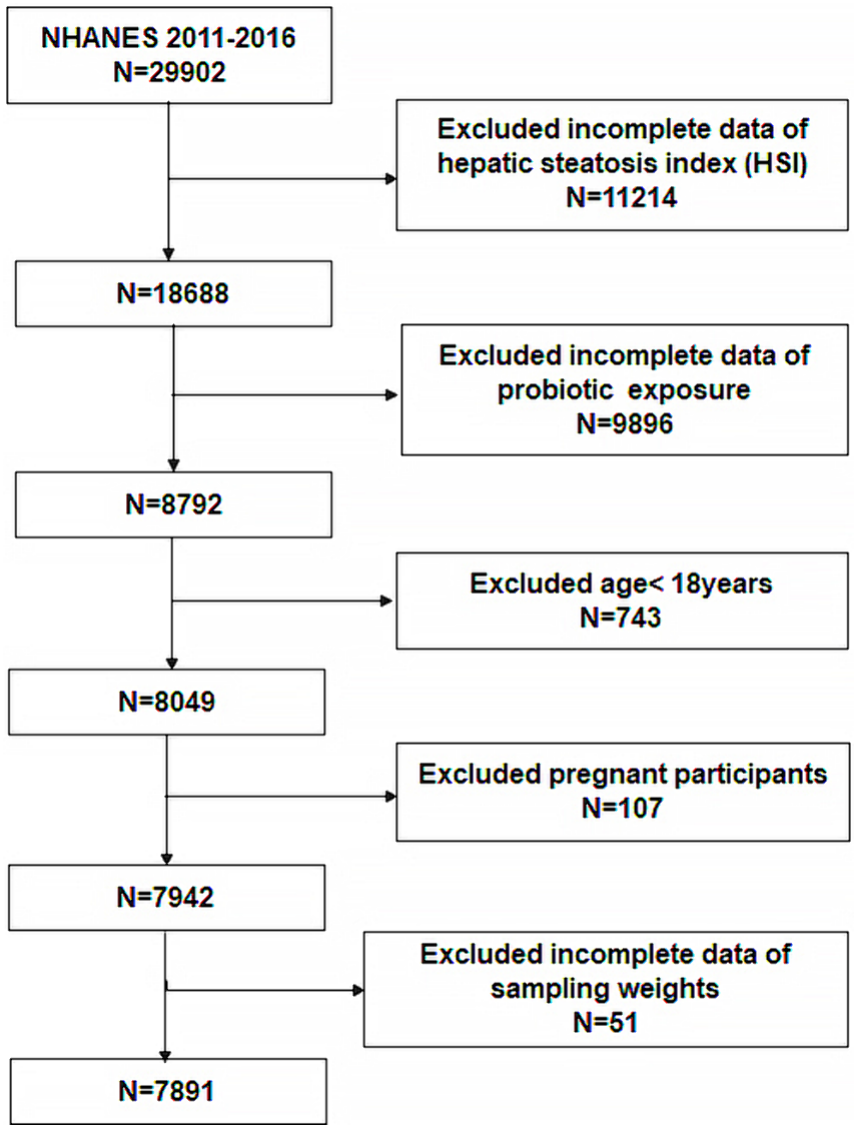
Flowchart of the participants selected from NHANES 2011–2016.

A total of 29,902 participants were included in the 2011–2016 survey cycle. Of these, we excluded 11,214 participants for whom the hepatic steatosis index (HSI) could not be calculated. 9,896 participants whose probiotic intake could not be determined were excluded. In addition, 743 participants age <18, 107 pregnant participants, and 51 participants whose data were without weight were all excluded. Eventually, 7,891 participants were enrolled (Figure 1).

### 2.2 Assessment and definition of probiotic yogurt consumption

Probiotic intake comes mainly from dietary sources (yogurt) and non-food sources (probiotic supplements). NHANES used an automated multiple-pass method to conduct recall dietary interviews to collect 24 h dietary intake (18). We utilized the standard deviation of the dietary data from both the in-person and telephone recalls for the NHANES participants from 2011 to 2016. We also utilized the Dietary Supplement Use 30-Day (DSQ), which evaluates the use of dietary supplements over the previous 30 days, to gauge exposure to probiotic supplements. List of probiotic supplements provided in the Supplementary Table S1. When a participant reported taking a probiotic supplement or yogurt (as a dietary source of probiotics) during the 24 h dietary recall or during the DSQ, it was taken into consideration that they had consumed probiotics. Probiotic supplements were defined as foods other than yogurt that included probiotics. MyPyramid converts yogurt consumption into the equivalent of a cup (19). The average value of yogurt from two interviews was used in this study. To assess consumption to probiotic supplements, text-mined was used to identify keywords for products with prebiotics, probiotics, and synbiotic elements in the dietary supplement questionnaire (DSQ) (20). Participants who consumed yogurt or probiotic supplements were probiotic consumption (21).

### 2.3 Covariates

Covariates that may influence HS include the following: age, gender, ratio of family income to poverty (PIR), race, education, smoke, physical activity level, diabetes mellitus (DM), hypertension, hyperlipidemia, body mass index (BMI), alanine aminotransferase (ALT), aspartate aminotransferase (AST), glycohemoglobin, total cholesterol (TC), triglycerides (TG), high-density lipoprotein-cholesterol (HDL-C), systolic blood pressure (SBP), diastolic blood pressure (DBP).

### 2.4 Definition of HS

HS was assessed using HSI. Currently, HSI is commonly used to evaluate MAFLD and HS (22, 23). Previous studies have established a good correlation between HSI and the degree of HS using examinations such as liver biopsy and ultrasonography (24, 25). HSI was calculated using the following formula: HSI = 8 × ALT/AST + BMI + 2 (if diabetes) + 2 (if female). HSI = 36 was considered the cut-off value for HS (26, 27).

### 2.5 MR analyses

Two-sample MR is considered a method of identifying the causal relationship between the phenotype of exposure and the outcome by using genetic variants for exposure as instrument variables (IV), which could make use of the accessible public dataset from large-sample genome-wide association studies (GWAS) for both “exposures” (as a risk factor) and “outcomes” (as a disease) and compensate for typical shortcomings of observational studies (28). The two-sample MR analysis was performed to investigate the causal relationship between yogurt intake and the risk of NAFLD. Publicly accessible GWAS databases were searched to find datasets meeting the criteria for exposure and outcomes. The main exposure data originated from an accessible GWAS dataset (GWAS ID: ukb-b-7753, including 64,949 Europeans, possessing 9,851,867 SNPs). Primary outcome data were sourced from an open GWAS dataset (GWAS ID: finn-b-NAFLD, encompassing 218,792 Europeans, with 16,380,466 SNPs). The GWAS database was searched for SNP selection using the assumptions stated above. To avoid linkage disequilibrium, all SNPs would be clumped using a stringent clump window (*r*^2^ = 0.001 and kb = 10,000). We chose 9 SNPs with a threshold of *p* < 5 × 10^6^ to detect possible associations with outcome confounders (29). F statistics were used to determine sample overlap and mild instrument bias. An *F* < 10 was deemed doubtful bias (30). The Inverse-variance weighted (IVW) method was used as the primary analysis method (31). MR-Egger, weighted median, simple mode, and weighted mode were employed as supplementary analysis methods (32). The MR Egger and IVW approaches were employed to evaluate heterogeneity, and pleiotropy was identified using the MR-Egger regression intercept. The leave-one-out sensitivity analysis was conducted to ascertain whether MR results were disproportionately influenced by a single SNP (33). The MR analysis in this study was conducted via the MR - Base platform^2^ (34).

### 2.6 Statistical analyses

Given the complex multistage cluster survey of the NHANES, all statistical analyses were performed using appropriate sampling weights according to NCHS guidelines to ensure national representation. Differences in the baseline characteristics of participants were compared using weighted Student’s t-test (continuous variables) or weighted chi-square test (categorical variables). Multivariate logistic regression was used to test the relationship between probiotic yogurt consumption and HS in three different models. For Model 1, no covariates were adjusted. For Model 2, age, race, education, and PIR were adjusted. For model 3, age, gender, PIR, race, education, smoking status, diabetes mellitus, hypertension, hyperlipidemia, ALT, AST, glycohemoglobin, TC, TG, HDL-c, SBP, and DBP were adjusted. Subgroup analyses were performed to investigate the relationship between probiotic yogurt consumption and HS in different subgroups. Stratification factors included age, gender, diabetes, hypertension, and hyperlipidemia. Interaction analysis was used to evaluate the heterogeneity of the relationship between the subgroups. To further analyze the relationship curve between food probiotics and HS, the curve relationship between yogurt intake and HS was demonstrated by restricted cubic spline (RCS). The “nhanesR” package was used to extract and analyze data. *p* < 0.05 was considered statistically significant.

## 3 Results

### 3.1 Baseline characteristics of participants

A total of 7,891 participants were enrolled, of whom 43.54% were male and 56.46% were female, with an average age of 51.37 years; 54.77% of participants were categorized as having HS. HS in patients was different with statistical significance of age, poverty income ratio (PIR), race, education, smoking status, physical activity level, DM, hypertension, hyperlipidemia, prebiotics, BMI, ALT, glycohemoglobin, TC, TG, HDL-c, SBP, and DBP (all *p* < 0.05). Gender and AST did not differ between participants with and without HS. The clinical and biochemical characteristics of the participants are shown in Table 1.

**Table 1:** Basic characteristics of participants (*n* = 7,891) in the NHANES 2011–2016.

Outcomes	Normal (*n* = 3,569)	Hepatic Steatosis (*n* = 4,322)	*p*-value
Age	49.15 ± 0.66	51.00 ± 0.47	0.01
Gender			0.47
Female	54.18 (52.19,56.16)	55.19 (53.38,56.99)	
Male	45.82 (43.84,47.81)	44.81 (43.01,46.62)	
PIR	3.34 ± 0.07	3.08 ± 0.06	<0.0001
Race			<0.0001
Mexican American	4.41 (3.30, 5.52)	8.34 (6.12,10.56)	
Non-Hispanic Black	5.92 (4.67, 7.17)	10.04 (7.87,12.21)	
Non-Hispanic White	75.31 (72.11,78.50)	68.92 (64.61,73.23)	
Other Hispanic	3.82 (2.61,5.03)	5.80 (4.25,7.34)	
Other Race	10.55 (8.58,12.51)	6.90 (5.75, 8.05)	
Education			<0.0001
High	74.82 (72.06,77.58)	66.81 (63.32,70.30)	
Medium	16.87 (14.97,18.76)	20.88 (18.70,23.05)	
Low	8.32 (6.84, 9.79)	12.32 (10.07,14.56)	
Smoke			<0.0001
Former	23.26 (21.03,25.49)	31.21 (29.10,33.31)	
Never	60.15 (57.73,62.56)	54.52 (52.23,56.82)	
Now	16.59 (14.38,18.81)	14.27 (12.80,15.73)	
Physical activity level			<0.0001
High	29.94 (27.23,32.65)	24.23 (22.12,26.33)	
Intermediate	31.05 (28.54,33.57)	25.28 (23.35,27.21)	
Low	24.55 (22.05,27.05)	26.83 (24.80,28.86)	
Unknown	14.45 (12.83,16.08)	23.66 (21.42,25.91)	
Diabetes Mellitus			<0.0001
Yes	5.44 (4.45, 6.43)	22.56 (20.44,24.68)	
No	94.56 (93.57,95.55)	77.44 (75.32,79.56)	
Hypertension			<0.0001
Yes	29.92 (27.37,32.47)	49.83 (47.49,52.18)	
No	70.08 (67.53,72.63)	50.17 (47.82,52.51)	
Hyperlipidemia			<0.0001
Yes	60.89 (57.70,64.08)	81.17 (79.43,82.91)	
No	39.11 (35.92,42.30)	18.83 (17.09,20.57)	
Prebiotics			<0.0001
Yes	40.24 (37.76,42.73)	33.41 (30.90,35.93)	
No	59.76 (57.27,62.24)	66.59 (64.07,69.10)	
BMI	23.9 ± 0.07	33.38 ± 0.16	<0.0001
ALT (U/L)	20.58 ± 0.30	28.50 ± 0.36	<0.0001
AST (U/L)	25.49 ± 0.41	25.99 ± 0.33	0.37
Glycohemoglobin (%)	5.40 ± 0.01	5.81 ± 0.03	<0.0001
TC (mmol/L)	4.94 ± 0.03	5.06 ± 0.03	0.002
TG (mmol/L)	1.39 ± 0.02	2.07 ± 0.04	<0.0001
HDL-c (mmol/L)	1.56 ± 0.01	1.29 ± 0.01	<0.0001
SBP (mmHg)	119.46 ± 0.45	124.85 ± 0.38	<0.0001
DBP (mmHg)	69.11 ± 0.33	71.44 ± 0.36	<0.0001

Mean ± SD was for continuous variables. The percentage (95% confidence interval) was for categorical variables. NHANES, National Health and Nutrition Examination Survey; PIR, poverty income ratio; BMI, body mass index; ALT, alanine aminotransferase; AST, aspartate aminotransferase; TC, total cholesterol; TG, triglycerides; HDL-c, high density lipoprotein-cholesterol; SBP, systolic blood pressure; DBP, diastolic blood pressure.

We further examined the relationship of probiotic yogurt consumption with patients diagnosed HS. In individuals with HS, 31.56% of participants consumed probiotics. Between HS with and without probiotic intake, significant statistical differences were observed in gender, PIR, race, education, smoking status, diabetes mellitus (DM), hypertension, BMI, glycohemoglobin, TC, TG, and HDL-c (all *p* < 0.05). Age, physical activity level, hyperlipidemia, HIS, ALT, AST, TC, SBP, and DBP did not differ between HS patients with and without consumption to probiotics. The clinical and biochemical characteristics of the HS patients are shown in Table 2.

**Table 2:** Basic characteristics of participants with HS with/without probiotic consumption in the NHANES 2011–2016.

Outcomes	HS without consumption to Probiotics (*n* = 2,958)	HS with consumption to Probiotics (*n* = 1,364)	*p*-value
Age	51.35 ± 0.44	50.30 ± 0.74	0.11
Gender			0.001
Female	51.88 (49.27,54.49)	61.77 (57.58,65.96)	
Male	48.12 (45.51,50.73)	38.23 (34.04,42.42)	
PIR	2.97 ± 0.06	3.30 ± 0.09	<0.001
Race			0.01
Mexican American	8.60 (6.12,11.08)	7.82 (5.47,10.18)	
Non-Hispanic Black	11.19 (8.63,13.74)	7.76 (5.87, 9.66)	
Non-Hispanic White	68.65 (63.76,73.54)	69.47 (64.89,74.04)	
Other Hispanic	5.71 (4.23,7.20)	5.96 (3.78,8.14)	
Other Race	5.85 (4.57, 7.14)	8.99 (6.74,11.24)	
Education			<0.0001
High	62.79 (58.95,66.62)	74.82 (70.46,79.19)	
Medium	23.08 (20.51,25.66)	16.48 (13.45,19.50)	
Low	14.13 (11.59,16.67)	8.70 (6.55,10.85)	
Smoking status			<0.001
Former	31.85 (29.34,34.36)	29.92 (25.65,34.19)	
Never	51.59 (49.11,54.07)	60.37 (56.12,64.62)	
Now	16.56 (14.54,18.57)	9.70 (7.33,12.08)	
Physical activity level			0.47
High	24.84 (22.09,27.59)	23.01 (19.40,26.62)	
Intermediate	24.32 (22.27,26.36)	27.20 (23.31,31.09)	
Low	26.73 (24.23,29.24)	27.02 (24.05,29.99)	
Unknown	24.11 (21.84,26.38)	22.77 (19.32,26.22)	
Diabetes Mellitus			0.04
Yes	23.83 (21.55,26.12)	20.03 (16.75,23.31)	
No	76.17 (73.88,78.45)	79.97 (76.69,83.25)	
Hypertension			<0.001
Yes	53.49 (50.99,55.98)	42.55 (37.81,47.29)	
No	46.51 (44.02,49.01)	57.45 (52.71,62.19)	
Hyperlipidemia			0.63
Yes	81.45 (79.38,83.53)	80.61 (77.73,83.49)	
No	18.55 (16.47,20.62)	19.39 (16.51,22.27)	
HSI	43.63 ± 0.20	43.14 ± 0.29	0.13
BMI	33.57 ± 0.17	32.99 ± 0.26	0.03
ALT (U/L)	28.39 ± 0.44	28.73 ± 0.77	0.72
AST (U/L)	25.85 ± 0.37	26.26 ± 0.68	0.61
Glycohemoglobin (%)	5.84 ± 0.03	5.75 ± 0.04	0.04
TC (mmol/L)	5.04 ± 0.03	5.11 ± 0.05	0.31
TG (mmol/L)	2.15 ± 0.05	1.90 ± 0.04	0.001
HDL-c (mmol/L)	1.27 ± 0.01	1.34 ± 0.02	<0.001
SBP (mmHg)	125.31 ± 0.40	123.94 ± 0.80	0.13
DBP (mmHg)	71.48 ± 0.41	71.37 ± 0.49	0.84

Mean ± SD was for continuous variables. The percentage (95% confidence interval) was for categorical variables. NHANES, National Health and Nutrition Examination Survey; PIR, poverty income ratio; BMI, body mass index; ALT, alanine aminotransferase; AST, aspartate aminotransferase; TC, total cholesterol; TG, triglycerides; HDL-c, high density lipoprotein-cholesterol; SBP, systolic blood pressure; DBP, diastolic blood pressure.

### 3.2 Relationship between HS and the consumption of probiotics, prebiotics, and yogurt

In univariate analysis, elevated age, PIR, former smoker status, DM, hypertension, hyperlipidemia, ALT, glycohemoglobin, TC, TG, lower HDL-c, SBP, and DBP were associated with a higher risk of HS (*p* < 0.05, Supplementary Table S2). To assess the independent effects of probiotic (yogurt) consumption on HS, three models were created after adjusting for potential confounding factors (Table 3). In the Model 1, multivariable logistic regression showed probiotics, yogurt consumption had significantly inverse interaction for HS (OR = 0.745, 95% CI: 0.664–0.836, *p* < 0.0001). After adjustments in the Model 2 for age, PIR, race, education, and physical activity level, a statistical significance remained in the relationship between probiotics, yogurt consumption and HS (adjusted OR = 0.82; 95% CI: 0.72–0.93, *p* = 0.003). After adjustment in the Model 3 for age, gender, race, poverty income ratio, education, smoking status, physical activity level, DM, hypertension, hyperlipidemia, ALT, AST, glycohemoglobin, TC, TG, HDL-c, SBP, DBP, probiotic and prebiotic supplements were an independent protective factor for HS (OR = 0.85, 95% CI: 0.73–0.99, *p* = 0.04). Compared to probiotic supplements, yogurt intake plays a more significant role in reducing HS (Table 4).

**Table 3:** Relationship between probiotics, yogurt consumption and HS.

	OR	95% CI	*p*-value
Model 1^a^	0.745	0.745 (0.664,0.836)	<0.0001
Model 2^b^	0.82	0.82 (0.72,0.93)	0.003
Model 3^c^	0.85	0.85 (0.73,0.99)	0.04

OR, odds ratio; CI, confidence interval.

a Model 1 did not adjust for any confounding factors.

b Model 2 adjusted for age, race, education, poverty income ratio, physical activity level.

c Model 3 adjusted for age, gender, race, poverty income ratio, education, smoking status, physical activity level, DM, hypertension, hyperlipidemia, ALT, AST, glycohemoglobin, TC, TG, HDL-c, SBP, DBP.

**Table 4:** Relationship between probiotic, prebiotic, yogurt and HS.

	OR	95% CI	*p*-value
Probiotic	0.792	0.792 (0.584,1.074)	0.130
Prebiotic	0.961	0.961 (0.676,1.366)	0.820
Yogurt	0.675	0.675 (0.526,0.866)	0.003

### 3.3 Subgroup analysis and interaction analysis

**Figure 2:**
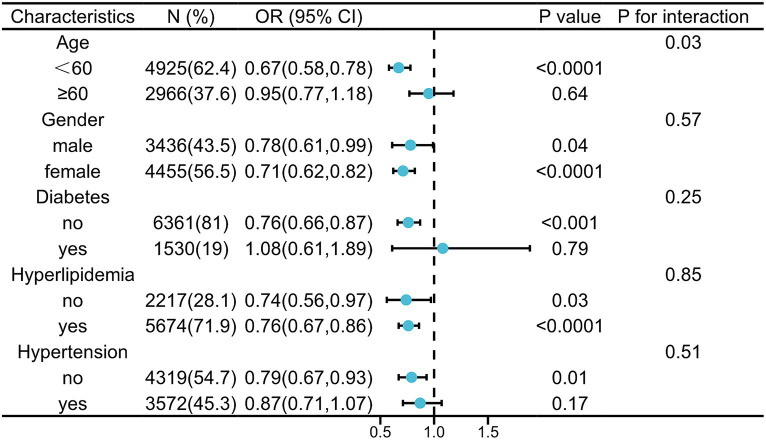
Subgroup analysis for the relationship between probiotic yogurt consumption and HS.

As for the subgroup stratified by age, gender, DM, hypertension, and hyperlipidemia, relationship with statistical significance was only observed in those participants with age <60 years, male or female, non-DM, non-hypertension, and hyperlipidemia. In addition, the interaction test showed no significant difference among gender, DM, hypertension, and hyperlipidemia, in the stratified subgroup analysis, indicating no significant dependence of them on the inverse relationship between probiotic consumption and HS (*p* for interaction >0.05). Conversely, that there was a significant dependence between age and this inverse relationship (*p* for interaction <0.05), indicating that the protective effect of probiotic yogurt against HS is more pronounced in individuals younger than 60 years. The results from subgroup analysis indicated that probiotic yogurt consumption was associated with a lower risk of HS (Figure 2).

### 3.4 MR analysis

**Figure 3:**
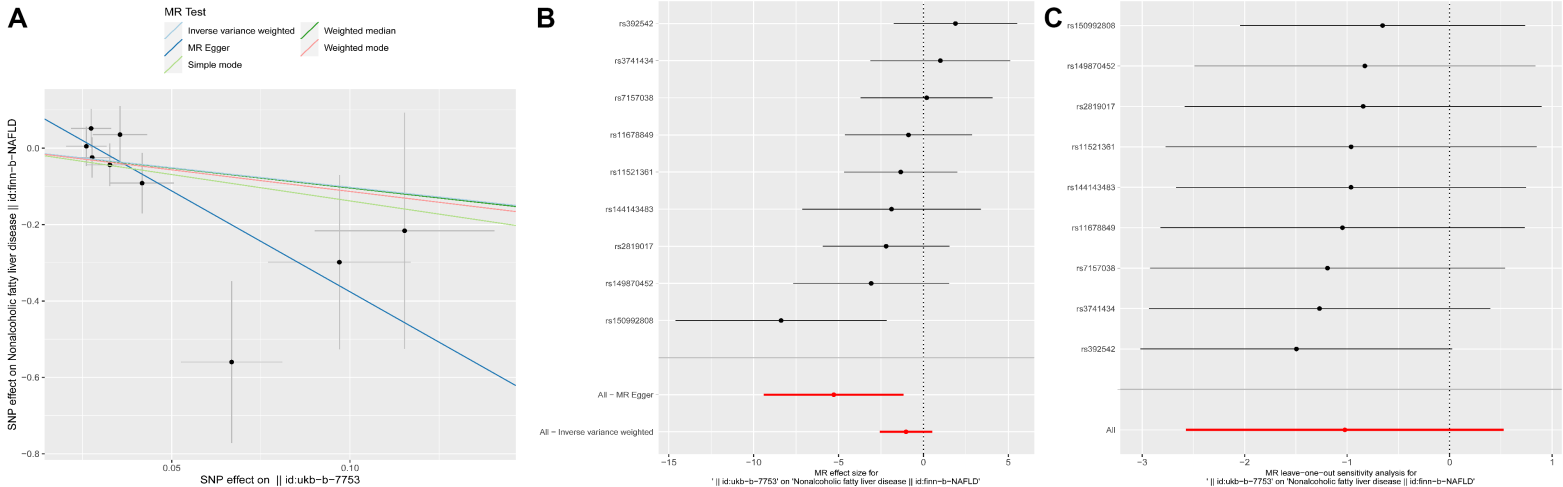
MR results of yogurt consumption and NAFLD. **(A)** Scatter plot of genetic correlations of yogurt intake and NAFLD using different MR methods. The slopes of line represent the causal effect of each method, respectively. **(B)** Forest plot of the causal effects of yogurt intake associated SNPs on NAFLD. **(C)** “Leave one out” analysis, the red lines are the analysis results of random effects IVW.

MR Egger analysis showed that there was a negative causal relationship between yogurt consumption and NAFLD (*p* = 0.04, OR = −5.282, Table 5 and Figure 3). The results of IVW, weighted median, simple mode, and weighted mode analysis indicated that there was a negative causal relationship between yogurt consumption and NAFLD, but it was not statistically significant (all *p* > 0.05, Table 5 and Figure 3). IVW and MR-Egger heterogeneity tests revealed consistent MR analysis results, evidencing no heterogeneity (MR Egger *p* = 0.5588; IVW *p* = 0.2354). The MR-Egger regression intercept results indicate that there is no pleiotropy in the MR analysis results (Egger regression intercept = 0.15, SE = 0.071, *p* = 0.0691).

**Table 5:** MR analysis of yogurt consumption and NAFLD.

Method	SNP	OR	SE	*p*-value
MR Egger	9	−5.282	2.102	0.04025
Weighted median	9	−1.043	0.9103	0.2521
Inverse variance weighted	9	−1.023	0.7909	0.1959
Simple mode	9	−1.381	1.423	0.36
Weighted mode	9	−1.133	1.178	0.3644

SE, standard error.

### 3.5 RCS analysis

**Figure 4:**
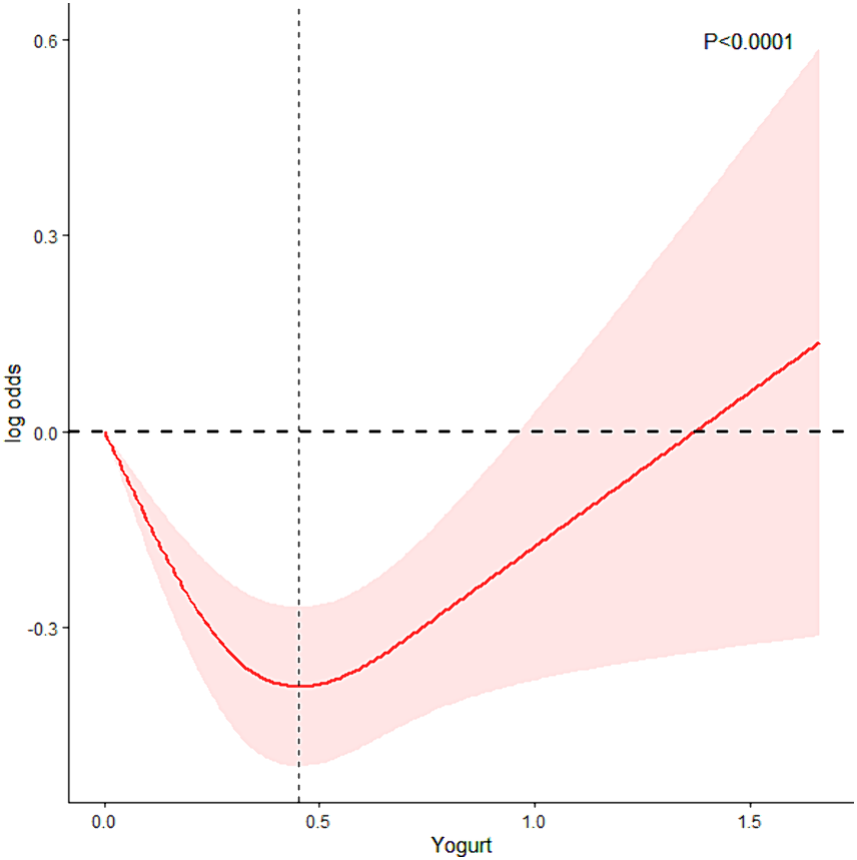
RCS analysis to explore the relationship between yogurt consumption and HS.

To explore the inconsistency between NHANES results and MR analysis outcomes, RCS was further employed for analysis. Figure 4 shows how RCS were used to create a flexible model and describe the unadjusted correlation between yogurt intake and HS. J-shaped link of yogurt with HS revealed a significant decrease in risk in the lower range of yogurt intake, which attained the lowest risk close to 0.4 cup. With yogurt intake greater than 0.4 cup, the probability of HS increased with the increase of yogurt intake (*p* for non-linearity <0.0001). The specified volume of 0.4 cups/day corresponds to approximately 95 milliliters/day (1 cup = 237 milliliters).

## 4 Discussion

We discovered an inverse relationship between probiotic yogurt consumption and HS in a large, nationally representative general population sample using NHANES data. The relationship remained after adjusting for several important predictors and covariates. To the best of our knowledge, this is also the first MR analysis to show the relationship between yogurt consumption and NAFLD. The current study is the first large-scale epidemiologic investigation of the relationship between probiotic or yogurt supplements and the prevalence of HS and the risk of HS development.

There is extensive evidence that gut microbes play a significant role in human disorders, interacting with numerous extra-intestinal organs such as the brain, cardiovascular, kidney, and skeletal systems (35). The gut and liver are anatomically and physiologically related, and their interaction with the gut microbiota forms the “gut-liver axis,” which has the possibility to adjust liver function and improve disease prognosis. Extensive research has been conducted to identify particular pathogenic species. HS has been associated with lower gut microbial diversity and the prevalence of *Coprococcus* and *Ruminococcus gnavus* in clinical trials (36). Both population-based and animal-based investigations of gastrointestinal microbes found that some microbial populations (*Bacteroides, Ruminococcus*, and so on) were dramatically altered in MAFLD patients compared to healthy controls (37). The severity of MAFLD was strongly connected to intestinal ecological dysregulation, with the abundance of Bacteroides associated with the progression of non-alcoholic steatohepatitis (NASH) and the abundance of *Ruminococcus* implicated in increased liver fibrosis (37). Loomba et al. revealed that NASH patients’ intestinal microbes varied in early and late liver fibrosis. Researchers were able to diagnose advanced liver fibrosis based on an increase in *Proteobacteria* and a considerable decrease in firmicutes in patients’ intestines (38).

With regard to mechanism, intestinal bacteria disrupt hepatic triglyceride metabolic homeostasis by raising endotoxin levels, influencing nutritional absorption, and affecting the amount and types of metabolites such as amino acids, fatty acids, and bile acids in the body (39). Several studies utilizing animal models of MAFLD have demonstrated the beneficial effects of probiotics as treatments for MAFLD (40–42). For instance, a study by Xin et al. revealed that mice fed a high-fat diet and treated with the probiotic *Lactobacillus johnsonii BS15* were able to prevent the onset of HS, a benefit attributed to reduced hepatic inflammation and oxidative stress (40). Similarly, Liang et al. conducted a trial indicating that mice with MAFLD, when fed a high-fat diet and supplemented with compound probiotics, showed improvements in gut dysbiosis and a decrease in hepatic lipid deposition (41). Several probiotics improved MAFLD liver oxidative stress and inflammatory liver damage mediated by nuclear factor-κ-gene binding (NF-κB) and c-Jun N-terminal kinase (JNK) in mouse models, with benefits related to insulin resistance (43, 44). In addition to animal experiments, several clinical trials using probiotics for MAFLD patients have been recorded. *Streptococcus thermophilus, Bifidobacterium breve, Bifidobacterium longum, Bifidobacterium infantis, Lactobacillus acidophilus, Lactobacillus plantarum, Lactobacillus paracasei*, and *Lactobacillus bulgaricus* (VSL #3) were the major clinical treatments for MAFLD (42, 44, 45). Besides, *conjugated linoleic acid* (CLA), a microbial metabolite generated by the VSL #3 probiotic, was related to MAFLD improvement (44, 45).

The clinical application of probiotics in liver illness is arising, with clinical and animal research demonstrating probiotics’ therapeutic potential in chronic liver disorders. Many studies have discovered that changes in the intestinal microbiota could affect liver function, systemic or hepatic inflammation, insulin resistance (IR), and fat accumulation, leading to obesity and, finally, liver injury and NASH progression (46). Indeed, despite the fact that probiotics have been used for decades to prevent or cure some illnesses, their efficacy in alleviating or combating HS has yet to be properly investigated. *Lactobacillus, Bifidobacteria*, and *Bacteroides bacteria* may replenish the gut microbial composition and decrease gastrointestinal endotoxemia, resulting in a strong anti-inflammatory response and the prevention of MAFLD development (47). The most prevalent probiotic bacteria are *Lactobacillus* and *Bifidobacterium*, followed by *Streptococcus, Escherichia, Enterococcus*, and *Bacillus*. Some fungal strains of *Saccharomyces* are also used as probiotics. Probiotic supplementation, alone or in conjunction with classic HS therapies, might then constitute a novel therapeutic method capable of restoring a normal gut bacterium, even if their synergic activity is unknown. In a double-blinded trial, Wong et al. observed that administering a probiotic formula containing *Lactobacillus plantarum, Lactobacillus bulgaricus, Lactobacillus acidophilus, Lactobacillus rhamnosus*, and *Bifidobacterium bifidum* to NASH patients over 6 months led to improved AST levels. This finding suggests a potential beneficial effect of probiotics on steatosis (48). In a 24 weeks study conducted by Bakhshimoghaddam et al. on 102 MAFLD patients, those randomized to consume 300 g of synbiotic yogurt containing 10^8 CFU of *Bifidobacterium animalis* showed a notable reduction in ultrasonography-graded MAFLD scores, including significant improvements in HS, AST, and ALT, in contrast to participants who consumed conventional yogurt or were in the control group (49). In an eight-week trial, MAFLD patients treated with the ‘Symbiter’ multi-probiotic, containing fourteen strains from five bacterial genera, saw significant HS reduction compared to controls, alongside lower liver enzymes and inflammatory markers (50). A meta-analysis of seven studies demonstrated that probiotic interventions in MAFLD significantly ameliorated AST, ALT levels, and the ultrasonic evaluation of HS (51). In fact, preclinical studies and randomized controlled trials are yet insufficient to show therapeutic efficacy in the therapy of HS, despite the fact that both are promising. Further research is needed to determine the most efficient probiotic strains that can be taken, the dosage that should be used, and the length of the treatment in order to better understand the specific function of the altered gut microbiota in the pathogenesis of HS.

There are some limitations in our study. First, we cannot determine temporality or causation because our study is cross-sectional in nature. Second, we have adjusted for socioeconomic status, comorbid conditions, and demographic factors in an effort to account for the healthy-user effect, which is a covariate factor but cannot be discounted (frequent probiotic/yogurt consumers have better and more balanced dietary habits, are healthier, and have fewer dietary restrictions than infrequent consumers). Third, another disadvantage is that while NHANES is mostly composed of healthy individuals, the severity of HS is low, making it difficult to establish substantial relationships with probiotic yogurt consumption due to ceiling impacts and a restricted range of pathology. Thus, these findings are just hypothesis-generating, and further prospective studies are needed to better define the relationship between probiotics consumption and HS.

## 5 Conclusion

In conclusion, our research revealed that using probiotics may provide a novel therapeutic method for controlling and treating HS. Further large-scale prospective studies are needed to validate our findings.

## Data availability statement

The datasets presented in this study can be found in online repositories. The names of the repository/repositories and accession number(s) can be found in the article/Supplementary material.

## Ethics statement

The studies involving humans were approved by the NCHS Research Ethics Review Board. The studies were conducted in accordance with the local legislation and institutional requirements. The participants provided their written informed consent to participate in this study.

## Author contributions

YS: Conceptualization, Data curation, Formal analysis, Investigation, Methodology, Project administration, Software, Supervision, Validation, Visualization, Writing – original draft, Writing – review & editing. WG: Conceptualization, Data curation, Formal analysis, Investigation, Methodology, Project administration, Software, Supervision, Validation, Visualization, Writing – original draft, Writing – review & editing. JW: Investigation, Software, Validation, Visualization, Writing – review & editing. SL: Formal analysis, Software, Supervision, Validation, Writing – review & editing. ZL: Software, Supervision, Validation, Writing – review & editing. YL: Conceptualization, Data curation, Funding acquisition, Investigation, Methodology, Project administration, Resources, Software, Supervision, Validation, Writing – original draft, Writing – review & editing.

## Footnotes

^1^ https://www.cdc.gov/nchs/nhanes

^2^ https://www.mrbase.org/

## References

[1] Festi D Colecchia A Sacco T Bondi M Roda E Marchesini G. Hepatic steatosis in obese patients: clinical aspects and prognostic significance. Obes Rev. (2004) 5:27–42. doi: 10.1111/j.1467-789x.2004.00126.x, PMID: 14969505

[2] Song Y Zhang J Wang H Guo D Yuan C Liu B. A novel immune-related genes signature after bariatric surgery is histologically associated with non-alcoholic fatty liver disease. Adipocytes. (2021) 10:424–34. doi: 10.1080/21623945.2021.1970341, PMID: 34506234 PMC8437528

[3] Eslam M Sanyal AJ George J. MAFLD: a consensus-driven proposed nomenclature for metabolic associated fatty liver disease. Gastroenterology. (2020) 158:1999–2014.e1. doi: 10.1053/j.gastro.2019.11.312, PMID: 32044314

[4] Eslam M Newsome PN Sarin SK Anstee QM Targher G Romero-Gomez M. A new definition for metabolic dysfunction-associated fatty liver disease: an international expert consensus statement. J Hepatol. (2020) 73:202–9. doi: 10.1016/j.jhep.2020.03.039, PMID: 32278004

[5] Hill C Guarner F Reid G Gibson GR Merenstein DJ Pot B. Expert consensus document. The international scientific Association for Probiotics and Prebiotics consensus statement on the scope and appropriate use of the term probiotic. Nat Rev Gastroenterol Hepatol. (2014) 11:506–14. doi: 10.1038/nrgastro.2014.66, PMID: 24912386

[6] Gibson GR Hutkins R Sanders ME Prescott SL Reimer RA Salminen SJ. Expert consensus document: the international scientific Association for Probiotics and Prebiotics (ISAPP) consensus statement on the definition and scope of prebiotics. Nat Rev Gastroenterol Hepatol. (2017) 14:491–502. doi: 10.1038/nrgastro.2017.75, PMID: 28611480

[7] Swanson KS Gibson GR Hutkins R Reimer RA Reid G Verbeke K. The international scientific Association for Probiotics and Prebiotics (ISAPP) consensus statement on the definition and scope of synbiotics. Nat Rev Gastroenterol Hepatol. (2020) 17:687–701. doi: 10.1038/s41575-020-0344-2, PMID: 32826966 PMC7581511

[8] Ford AC Harris LA Lacy BE Quigley EMM Moayyedi P. Systematic review with meta-analysis: the efficacy of prebiotics, probiotics, synbiotics and antibiotics in irritable bowel syndrome. Aliment Pharmacol Ther. (2018) 48:1044–60. doi: 10.1111/apt.15001, PMID: 30294792

[9] Scorletti E Afolabi PR Miles EA Smith DE Almehmadi A Alshathry A. Synbiotics Alter fecal microbiomes, but not liver fat or fibrosis, in a randomized trial of patients with nonalcoholic fatty liver disease. Gastroenterology. (2020) 158:1597–1610.e7. doi: 10.1053/j.gastro.2020.01.031, PMID: 31987796 PMC7613160

[10] Rong L Ch’ng D Jia P Tsoi KKF Wong SH Sung JJY. Use of probiotics, prebiotics, and synbiotics in non-alcoholic fatty liver disease: a systematic review and meta-analysis. J Gastroenterol Hepatol. (2023) 38:1682–94. doi: 10.1111/jgh.16256, PMID: 37409560

[11] Loman BR Hernández-Saavedra D An R Rector RS. Prebiotic and probiotic treatment of nonalcoholic fatty liver disease: a systematic review and meta-analysis. Nutr Rev. (2018) 76:822–39. doi: 10.1093/nutrit/nuy031, PMID: 30113661

[12] Schnabl B Brenner DA. Interactions between the intestinal microbiome and liver diseases. Gastroenterology. (2014) 146:1513–24. doi: 10.1053/j.gastro.2014.01.020, PMID: 24440671 PMC3996054

[13] Del Chierico F Nobili V Vernocchi P Russo A De Stefanis C Gnani D. Gut microbiota profiling of pediatric nonalcoholic fatty liver disease and obese patients unveiled by an integrated meta-omics-based approach. Hepatology. (2017) 65:451–64. doi: 10.1002/hep.28572, PMID: 27028797

[14] Lirussi F Mastropasqua E Orando S Orlando R. Probiotics for non-alcoholic fatty liver disease and/or steatohepatitis. Cochrane Database Syst Rev. (2007) 2010:CD005165. doi: 10.1002/14651858.CD005165.pub2, PMID: 17253543 PMC8865955

[15] Tarantino G Finelli C. Systematic review on intervention with prebiotics/probiotics in patients with obesity-related nonalcoholic fatty liver disease. Future Microbiol. (2015) 10:889–902. doi: 10.2217/fmb.15.13, PMID: 26000656

[16] Ebrahimi-Mousavi S Alavian SM Sohrabpour AA Dashti F Djafarian K Esmaillzadeh A. The effect of daily consumption of probiotic yogurt on liver enzymes, steatosis and fibrosis in patients with nonalcoholic fatty liver disease (NAFLD): study protocol for a randomized clinical trial. BMC Gastroenterol. (2022) 22:102. doi: 10.1186/s12876-022-02176-2, PMID: 35255811 PMC8899796

[17] Davey Smith G Hemani G. Mendelian randomization: genetic anchors for causal inference in epidemiological studies. Hum Mol Genet. (2014) 23:R89–98. doi: 10.1093/hmg/ddu328, PMID: 25064373 PMC4170722

[18] Cifelli CJ Agarwal S Fulgoni VL 3rd. Association of Yogurt Consumption with nutrient intakes, nutrient adequacy, and diet quality in American children and adults. Nutrients. (2020) 12:3435. doi: 10.3390/nu12113435, PMID: 33182430 PMC7696083

[19] Keast DR Hill Gallant KM Albertson AM Gugger CK Holschuh NM. Associations between yogurt, dairy, calcium, and vitamin D intake and obesity among U.S. children aged 8-18 years: NHANES, 2005-2008. Nutrients. (2015) 7:1577–93. doi: 10.3390/nu7031577, PMID: 25742042 PMC4377867

[20] O’Connor LE Gahche JJ Herrick KA Davis CD Potischman N Vargas AJ. Nonfood prebiotic, probiotic, and Synbiotic use has increased in US adults and children from 1999 to 2018. Gastroenterology. (2021) 161:476–486.e3. doi: 10.1053/j.gastro.2021.04.037, PMID: 33895169

[21] Lau E Neves JS Ferreira-Magalhães M Carvalho D Freitas P. Probiotic ingestion, obesity, and metabolic-related disorders: results from NHANES, 1999-2014. Nutrients. (2019) 11:71482. doi: 10.3390/nu11071482, PMID: 31261830 PMC6683043

[22] Lee JH Kim D Kim HJ Lee CH Yang JI Kim W. Hepatic steatosis index: a simple screening tool reflecting nonalcoholic fatty liver disease. Dig Liver Dis. (2010) 42:503–8. doi: 10.1016/j.dld.2009.08.002, PMID: 19766548

[23] Song Y Guo W Liu S Li Z Guo D Li Y. Individuals undergoing bariatric surgery ameliorate hepatic steatosis: evidence from NHANES 2015-2018. Obes Surg. (2022) 32:3811. doi: 10.1007/s11695-022-06284-6, PMID: 36125696

[24] Chon YE Jung KS Kim SU Park JY Park YN Kim DY. Controlled attenuation parameter (CAP) for detection of hepatic steatosis in patients with chronic liver diseases: a prospective study of a native Korean population. Liver Int. (2014) 34:102–9. doi: 10.1111/liv.12282, PMID: 24028214

[25] Shih KL Su WW Chang CC Kor CT Chou CT Chen TY. Comparisons of parallel potential biomarkers of 1H-MRS-measured hepatic lipid content in patients with non-alcoholic fatty liver disease. Sci Rep. (2016) 6:24031. doi: 10.1038/srep24031, PMID: 27079922 PMC4832180

[26] Kim D Manikat R Cholankeril G Ahmed A. Endogenous sex hormones and nonalcoholic fatty liver disease in US adults. Liver Int. (2024) 44:460–71. doi: 10.1111/liv.15786, PMID: 38010926

[27] Wu Z Ouyang T Liu H Cao L Chen W. Perfluoroalkyl substance (PFAS) exposure and risk of nonalcoholic fatty liver disease in the elderly: results from NHANES 2003-2014. Environ Sci Pollut Res Int. (2023) 30:64342–51. doi: 10.1007/s11356-023-26941-2, PMID: 37067713

[28] Skrivankova VW Richmond RC Woolf BAR Yarmolinsky J Davies NM Swanson SA. Strengthening the reporting of observational studies in epidemiology using Mendelian randomization: the STROBE-MR statement. JAMA. (2021) 326:1614–21. doi: 10.1001/jama.2021.18236, PMID: 34698778

[29] Zou XL Wang S Wang LY Xiao LX Yao TX Zeng Y. Childhood obesity and risk of stroke: a Mendelian randomisation analysis. Front Genet. (2021) 12:727475. doi: 10.3389/fgene.2021.727475, PMID: 34868204 PMC8638161

[30] Pierce BL Ahsan H Vanderweele TJ. Power and instrument strength requirements for Mendelian randomization studies using multiple genetic variants. Int J Epidemiol. (2011) 40:740–52. doi: 10.1093/ije/dyq151, PMID: 20813862 PMC3147064

[31] Burgess S Dudbridge F Thompson SG. Combining information on multiple instrumental variables in Mendelian randomization: comparison of allele score and summarized data methods. Stat Med. (2016) 35:1880–906. doi: 10.1002/sim.6835, PMID: 26661904 PMC4832315

[32] Xu J Zhang S Tian Y Si H Zeng Y Wu Y. Genetic causal association between Iron status and osteoarthritis: a two-sample Mendelian randomization. Nutrients. (2022) 14:3683. doi: 10.3390/nu14183683, PMID: 36145059 PMC9501024

[33] Zheng J Baird D Borges MC Bowden J Hemani G Haycock P. Recent developments in Mendelian randomization studies. Curr Epidemiol Rep. (2017) 4:330–45. doi: 10.1007/s40471-017-0128-6, PMID: 29226067 PMC5711966

[34] Hemani G Zheng J Elsworth B Wade KH Haberland V Baird D. The MR-base platform supports systematic causal inference across the human phenome. eLife. (2018) 7:7. doi: 10.7554/eLife.34408, PMID: 29846171 PMC5976434

[35] Singhvi N Gupta V Gaur M Sharma V Puri A Singh Y. Interplay of human gut microbiome in health and wellness. Indian J Microbiol. (2020) 60:26–36. doi: 10.1007/s12088-019-00825-x, PMID: 32089571 PMC7000599

[36] Alferink LJM Radjabzadeh D Erler NS Vojinovic D Medina-Gomez C Uitterlinden AG. Microbiomics, metabolomics, predicted metagenomics, and hepatic steatosis in a population-based study of 1,355 adults. Hepatology. (2021) 73:968–82. doi: 10.1002/hep.31417, PMID: 32530501

[37] Boursier J Mueller O Barret M Machado M Fizanne L Araujo-Perez F. The severity of nonalcoholic fatty liver disease is associated with gut dysbiosis and shift in the metabolic function of the gut microbiota. Hepatology. (2016) 63:764–75. doi: 10.1002/hep.28356, PMID: 26600078 PMC4975935

[38] Loomba R Seguritan V Li W Long T Klitgord N Bhatt A. Gut microbiome-based metagenomic signature for non-invasive detection of advanced fibrosis in human nonalcoholic fatty liver disease. Cell Metab. (2017) 25:1054–1062.e5. doi: 10.1016/j.cmet.2017.04.001, PMID: 28467925 PMC5502730

[39] Loomba R Friedman SL Shulman GI. Mechanisms and disease consequences of nonalcoholic fatty liver disease. Cell. (2021) 184:2537–64. doi: 10.1016/j.cell.2021.04.015, PMID: 33989548 PMC12168897

[40] Xin J Zeng D Wang H Ni X Yi D Pan K. Preventing non-alcoholic fatty liver disease through *Lactobacillus johnsonii* BS15 by attenuating inflammation and mitochondrial injury and improving gut environment in obese mice. Appl Microbiol Biotechnol. (2014) 98:6817–29. doi: 10.1007/s00253-014-5752-1, PMID: 24811405

[41] Liang Y Liang S Zhang Y Deng Y He Y Chen Y. Oral Administration of Compound Probiotics Ameliorates HFD-induced gut microbe Dysbiosis and chronic metabolic inflammation via the G protein-coupled receptor 43 in non-alcoholic fatty liver disease rats. Probiotics Antimicrob Proteins. (2019) 11:175–85. doi: 10.1007/s12602-017-9378-3, PMID: 29353414

[42] Borrelli A Bonelli P Tuccillo FM Goldfine ID Evans JL Buonaguro FM. Role of gut microbiota and oxidative stress in the progression of non-alcoholic fatty liver disease to hepatocarcinoma: current and innovative therapeutic approaches. Redox Biol. (2018) 15:467–79. doi: 10.1016/j.redox.2018.01.009, PMID: 29413959 PMC5975181

[43] Li Z Yang S Lin H Huang J Watkins PA Moser AB. Probiotics and antibodies to TNF inhibit inflammatory activity and improve nonalcoholic fatty liver disease. Hepatology. (2003) 37:343–50. doi: 10.1053/jhep.2003.50048, PMID: 12540784

[44] Velayudham A Dolganiuc A Ellis M Petrasek J Kodys K Mandrekar P. VSL#3 probiotic treatment attenuates fibrosis without changes in steatohepatitis in a diet-induced nonalcoholic steatohepatitis model in mice. Hepatology. (2009) 49:989–97. doi: 10.1002/hep.22711, PMID: 19115316 PMC3756672

[45] Chang B Sang L Wang Y Tong J Zhang D Wang B. The protective effect of VSL#3 on intestinal permeability in a rat model of alcoholic intestinal injury. BMC Gastroenterol. (2013) 13:151. doi: 10.1186/1471-230x-13-151, PMID: 24138544 PMC4016537

[46] Cani PD Amar J Iglesias MA Poggi M Knauf C Bastelica D. Metabolic endotoxemia initiates obesity and insulin resistance. Diabetes. (2007) 56:1761–72. doi: 10.2337/db06-1491, PMID: 17456850

[47] Xue L He J Gao N Lu X Li M Wu X. Probiotics may delay the progression of nonalcoholic fatty liver disease by restoring the gut microbiota structure and improving intestinal endotoxemia. Sci Rep. (2017) 7:45176. doi: 10.1038/srep45176, PMID: 28349964 PMC5368635

[48] Wong VW Won GL Chim AM Chu WC Yeung DK Li KC. Treatment of nonalcoholic steatohepatitis with probiotics. A proof-of-concept study. Ann Hepatol. (2013) 12:256–62. doi: 10.1016/S1665-2681(19)31364-X, PMID: 23396737

[49] Bakhshimoghaddam F Shateri K Sina M Hashemian M Alizadeh M. Daily consumption of Synbiotic yogurt decreases liver steatosis in patients with nonalcoholic fatty liver disease: a randomized controlled clinical trial. J Nutr. (2018) 148:1276–84. doi: 10.1093/jn/nxy088, PMID: 29931231

[50] Kobyliak N Abenavoli L Mykhalchyshyn G Kononenko L Boccuto L Kyriienko D. A multi-strain probiotic reduces the fatty liver index, cytokines and aminotransferase levels in NAFLD patients: evidence from a randomized clinical trial. J Gastrointestin Liver Dis. (2018) 27:41–9. doi: 10.15403/jgld.2014.1121.271.kby, PMID: 29557414

[51] SL A VR D Manohar T AL A. Role of probiotics in the treatment of nonalcoholic fatty liver disease: a meta-analysis. Euroasian J Hepatogastroenterol. (2017) 7:130–7. doi: 10.5005/jp-journals-10018-1233, PMID: 29201794 PMC5670255

